# Supplementary material for: Heat adaptation of phage T7 under an extended genetic code
Source: Virus Evol. 2021 Dec 1;7(2):veab100. doi: 10.1093/ve/veab100 (PMC8923235; doi:10.1093/ve/veab100)
Supplement: veab100_Supp [file veab100_supp.zip › Sequencing_Analysis.zip!/Sequencing_Analysis/A1/evidence/SNP_3.html]

BRESEQ :: Evidence


| Predicted mutation | | | | | | |
| --- | --- | --- | --- | --- | --- | --- |
| evidence | seq id | position | mutation | annotation | gene | description |
| RA | NC\_001604.1 | 18,406 | G→A | intergenic (–/–) | – / – | –/– |

| Read alignment evidence... | | | | | | | | | | | |
| --- | --- | --- | --- | --- | --- | --- | --- | --- | --- | --- | --- |
|  | seq id | position | | ref | new | freq | score (cons/poly) | reads | annotation | genes | product |
| \* | NC\_001604.1 | 18,406 | 0 | G | A | 99.6% | 9510.7 / NA | 4118 | intergenic (–/–) | *–/–* | –/– |
| Reads supporting (aligned to +/- strand):  **ref** base G (2/3);  **major** base A (2573/1516);  **minor** base C (15/2);  **total** (2594/1524) | | | | | | | | | | | |
| Fisher's exact test for biased strand distribution *p*-value = 4.10e-02 | | | | | | | | | | | |
| Kolmogorov-Smirnov test that lower quality scores support variant *p*-value = 1.00e+00 | | | | | | | | | | | |

Only 100 of 4122 total aligned reads displayed.  

`ATGGTTACTCAGGGATTGCTGGATGGGGTGATACCGCCGAGGACTTCTTGAATAACCCGTTCATAACCGAGCCTAAAACGTCTGTGCTTAAGTCCGGTAAGAACAAAGGCCAAGAGGTTACTAAATGGGTTAAACGCGACCCTGAGCCTCATGAGACGCTTTGGGACTGCATTAAGTCCATTGGCGCGAAGGCTGGTATGACCGAAGAGGATATTATCAAGCAGGGCCAAATGGCTCGAATCCTACGGTTCAACGAGTACAACTTTATTGACAAGGAGATTTACCTG‑TGGAGACCGTAGCGTATATTGGTCTGGGTCTTTGTGTTCTCGGAGTGTGCCTCATTTCGTGGGGCCTTTGGGACTTAGCCAGAATAATCAAGTCGTTACACGACACTAAGTGATAAACTCAAGGTCCCTAAATTAATACGACTCACTATAGGGAGATAGGGGCCTTTACGATTATTACTTTAAGATTTAACTCTAAGAGGAATCTTTATTATGTTAACACCTATTAACCAATTACTTAAGAACCCTAACGATATTCCAGATGTACCTCGTGCAACCGCTGAGTATCTACAGGTTCGATTCAACTATGCGTAC  >  NC_001604.1/18108‑18706`  

                                                                                                                                                                                                                                                                                                           `|`                                                                                                                                                                                                                                                                                                              
 `tGGTTACTCAGGGATTGCTGGATGGGGTGATACCGCCGAGGACTTCTTGAATAACCCGTTCATAACCGAGCCTAAAACGTCTGTGCTTAAGTCCGGTAAGAACAAAGGCCAAGAGGTTACTAAATGGGTTAAACGCGACCCTGAGCCTCATGAGACGCTTTGGGACTGCATTAAGTCCATTGGCGCGAAGGCTGGTATGACCGAAGAGGATATTATCAAGCAGGGCCAAATGGCTCGAATCCTACGGTTCAACGAGTACAACTTTATTGACAAGGAGATTTACCTG‑TGGAGACCGTAACGt                                                                                                                                                                                                                                                                                                           <  1:394798/301‑1 (MQ=255)`  
 `ggtataatCAGGGATGGCTGGATGGGGTGATGCCGCCGAGGCATTCTTGAATAACCCCTTCATAACCGATCATAAAACGTCTGTGCTTAAGTACGGTGAGAACAAACGCCAAGATGGTACTAGCTGGGTTAGACGCGACCGAGAGCCACATGTGACGCTTTGGGACTGTATTTAGTCCATTGGAGCGAAGGCTTGTATGACCGAAGAGGATATTATCAAGCAGGGCCAAATGGCTCGAATCCTACGGTTCAACGAGTACAGCTTTATTGACAAGGAGATTTACCTG‑TGGAGACCGTAACGt                                                                                                                                                                                                                                                                                                           <  1:441273/294‑1 (MQ=255)`  
 `gggTTACTAAGGGAGTGCTGGATGGGGTGATACCGCCGAGGACTTCTTGAATAACCCGTTCATAACCGAGCCTAAAACGTCTGTGCTTAAGTCCGGTAAGAACAAAGGACAAGAGGTTACTAAATGGGTTAAACGCGACCCTGAGCCTCATGAGACGCTTTGGGACTGCATTAAGTCCATTGGCGCGAAGGCTGGTATGACCGAAGAGGATATTATCAAGCAGGGCCAAATGGCTCGAAGCCTACGGTTCAACGAGTACAACTTTATTGACAAGGAGATTTACCTG‑TGGAGACCGTAACGt                                                                                                                                                                                                                                                                                                           <  1:82100/300‑1 (MQ=255)`  
   `gttaagcAGGGATTGCTGGATGGGGTTATACCGCCGAGGACTTCTTGAATAACCCGTTCATAACCGAGCCTAAAACGTCTGTGCTTAAGTCCGGTAAGAACAAAGGCCAAGAGGTTACTAAATGGGTTAAACGCGACCCTGAGCCTCATGAGACGCTTTGGGACTGCATTAAGTCCATTGGCGCGAAGGCTGGTATGACCGAAGAGGATATTATCAAGCAGGGCCAAATGGCTCGAATCCTACGGTTCAACGAGTACAACTTTATTGACAAGGAGATTTACCTG‑TGGAGACCGTAACGtat                                                                                                                                                                                                                                                                                                         <  1:221698/295‑1 (MQ=255)`  
     `gagatgtgtataagatacaggggTGATACCGCCGAGGACTTCTTGAATAACCAGTTCTTAACCGATCCTAAAACTTCTGTGATTAAGTCCGGGAATAACAAAGGCCAAGAGGTTACTAAATGGGTTAAACGCGACCCTGAGCCTCATGAGTCGCTTTGGGACTGCATTAAGTCCATTGGCGCGAAGGCTGGTATGACCGAAGAGGATATTATCAAGCAGGGCCAAATTGCTCGAATCCTACGGTTCAACGAGTACAACTTTATTGACAAGGGGATTTACCTG‑TGGAGACCGTAACGTatat                                                                                                                                                                                                                                                                                                       <  1:296509/282‑1 (MQ=255)`  
      `aCTCAGGGATTGCTGGATGGGGTGATACCGCCGAGGACTTCTTGAATAACCCGTTCATAACCGAGCCTAAAACGTCTGTGCTTAAGTCCGGTAAGAACACAGGCCAAGAGGTTACTAAATGGGTTAAACGCGACCCTGAGCCTCATGAGACGCTTTGGGACTGCATTAAGTCCATTGGCGCGAAGGCTGGTATGACCGAAGAGGATATTATCAAGCAGGGCCAAATGGCTCGAATCCTACGGTTCAACGAGTACAACTTTATTGACAAGGAGATTTACCTG‑TGGAGACCTTAACGTATAtt                                                                                                                                                                                                                                                                                                      >  1:341347/1‑301 (MQ=255)`  
       `agagacagttGCTGGATGGGGTGATACCGCCGAGGACTTCTTGAATAACCCGTTCATAACCGAGCCTATAACGTCTGTGCTTAAGTCCGGTAAGAACAAAGGCCAAGAGGTTACTAAATGGGTTAAACGCGACCCTGAGCCTCATGAGACGCTTTGGGACTGCATTAAGTCCATTGGCCCGAAGGCTGGTATGACCGAAGAGGATATTATCAAGCAGGGCCAAATGGCTCGAATCCTACGGTTCAACGAGTACAACTTTATTGACAAGGAGATTTACCTG‑TGGAGACCGTAACGTATATTg                                                                                                                                                                                                                                                                                                     <  1:420949/293‑1 (MQ=255)`  
        `cggagatgttgataagagacagATACCGACGAAGACTTCTTGAATAACCCGTTCATAACCTAGCCGAAAACGTTGGTGCTGAAGTCCGGTAAGAACAAAGGCCAAGAGGTTACTAAATGGGTTAAAGGCGACCCTTAGCCTCATGGGACGCTTTGGGAATGCATTAAGTCCATTGTGGCGAAGGGCGGTATGTCCGAAAAGGTTATTATCAAGCAGGGCCAAATGGCTCGAATCCTACGGTTCCACGCGTACAACTTTATTGACAAGGAGATTTACCTG‑TGCATTCCGTAACGTATATTgg                                                                                                                                                                                                                                                                                                    <  1:314274/280‑1 (MQ=255)`  
          `gtgtataagagagaggggTGATACAGCCGAGGACTTCATGAATAACCCGTTCATAAACGAGCCTAAAACGTCTGTGCTTAAGTACGGGAAGAACAAAGGCCAAGAGGTTAATAAATGGGTTAAACGAGACCCTGAGCCTAAAGAGACGCTTTGGGACTGCATTAAGTCCATTGGCGCGAAGGCTGGTAGGACCGAAGATGATATTATCAAGCAGGGCCAAATGGCGAGAATCCTACGGTTCAACGAGTACAACTTTATTGACAAGGAGATTTACCTG‑TGGAGACCGTAACGTATATTGGTc                                                                                                                                                                                                                                                                                                  <  1:504668/287‑1 (MQ=255)`  
           `gggATTGCTGGATGGGGTGATACCGCCGAGGACTTCTTGAATAACCCGTTCATAACCGAGCCTAAAACGTCTGTGCTTAAGTCCGGTAAGAACGAAGGCCAAGAGGTTACTAAATGGGTTAAACGCGACCCTGAGCCTCATGAGACGCTTTGGGACTGCATTAAGTCCATTGGCGCGAAGGCTGGTATGACCGAAGAGGATATTATCAAGCAGGGCCAAATGGCTCGAATCCTACGGTTCAACGAGTACAACTTTATTGACAAGGAGATTTACCTG‑TGGAGACCGTAACGTATATTGGTCt                                                                                                                                                                                                                                                                                                 >  1:482920/1‑301 (MQ=255)`  
               `ttGCTGTATGGGGTGATACCGCCGAGGACTTCTTGAATAACCCGTTCATAACCGAGCCTAAAACGTCTGTGCTTAAGTCCGGTAAGAACAAAGGCCAAGAGGTTACTAAATGGGTTAAACGCGACCCTGAGCCTCATAAGACGCTTTGGGACTGCATTAAGTCCATTGGCGCGCAGGCTGGTATGACCGAAGAGGATATTATCAAGCAGGGCCAAATGGCTCGAATCCTACGGTTCCACGAGTACAACTTTATTGACAAGGAGATTTACCTG‑TGGAGACCGTAACGTATATTggctgtctc                                                                                                                                                                                                                                                                                             >  1:230436/1‑294 (MQ=255)`  
               `ttGCTGGATGGGGTGATACCGCCGAGGACTTCTTGAATAACCCGTTCATAACCGAGCCTAAAACGTCTGTGCTTAAGTCCGGTAAGAACAAAGGCCAAGAGGTTACTAAATGGGTTAAACGCGACCCTGAGTCTCATGAGACGCTTTGGGACTGCATTAAGTCTATTGGCGCGAAGGCTGGTATGACCGAAGAGGATATTATCAAGCAGGGCCAAATGGCTCGAATCCTACGGTTCAACGAGTACAACTTTATTGACAAGGAGATTTACCTG‑TGGAGACCGTAACGTATATTGGTCTGGGt                                                                                                                                                                                                                                                                                             <  1:241373/301‑1 (MQ=255)`  
               `ttGCTGGATGGGGTGATACCGCCGAGGACTTCTTGAATAACCCGTTCATAACCGAGCCTAAAACGTCTGTGCTTAAGTCCGGTAAGAACAAAGGCCAAGAGGTTACTAAATGGGTTAAACGCGACCCTGAGCCTCATGAGACGCTTTGGGACTGCATTAAGTCCATTGGCGCGAAGGCTGGTATGACCGAAGAGGATATTATCAAGCAGGGCCAAATGGCTCGAATCCTACGGTTCAACGAGTACAACTTTATTGACAAGGAGATTTACCTG‑TGGAGACCGTAACGTATATTGGTCTGGGt                                                                                                                                                                                                                                                                                             >  1:119720/1‑301 (MQ=255)`  
                 `gCTGGATGGGGTGATACCGCCGAGGACTTCTTGAATAACCCGTTCATAACCGAGCCTAAAACGTCTGTGCTTAAGTCCGGTAAGAACAAAGGCCAAGAGGTTACTAAATGGGTTAAACGCGACCCTGAGCCTCATGAGACGCTTTGGGACTGCATTAAGTCCATTGGCGCGAAGGCTGGTATGACCGAAGAGGATATTATCAAGCAGGGCCAAATGGCTCGAATCCTACGGTTCAACGAGTACAACTTTATTGACAAGGAGATTTACCTG‑TGGAGACCGTAACGTATATTGGTCTTGGACt                                                                                                                                                                                                                                                                                           >  1:489642/1‑301 (MQ=255)`  
                 `gCTGGATGGGGTGATACCGCCGAGGACTTCTTGAATAACCCGTTCATAACCGAGCCTAAAACGTCTGTGCTTAAGTCCGGTAAGAACAAAGGCCAAGAGGTTACTAAATGGGTTAAACGCGACCCTGAGCCTCATGAGACGCTTTGGGACTGCATTAAGTCCATTGGCGCGAAGGCTGGTATGACCGAAGAGGATATTATCAAGCAGGGCCAAATGGCTCGAATCCTACGGTTCAACGAGTACAACTTTATTGACAAGGAGATTTACCTG‑TGGAGACCGTAACGTATATTGGTCTGGGTCt                                                                                                                                                                                                                                                                                           >  1:150807/1‑301 (MQ=255)`  
                    `ggATGGGGTGATACCGCCGAGGACTTCTTGAATAACCCGTTCATAACCGAGCCTAAAACGTCTGTGCTTAAGTCCGGTAAGAACAAAGGCCAAGAGGTTACTAAATGGGTTAAACGCGACCCTGAGCCTCATGAGACGCTTTGGGACTGCATTAAGTCCATTGGCGCGAAGGCTGGTATGACCGAAGAGGATATTATCAAGCAGGGCCAAATGGCTCGAATCCTACGGTTCAACGAGTACAACTTTATTGACAAGGAGATTTACCTG‑TGGAGACCGTAACGtctgtctcttatacacatct                                                                                                                                                                                                                                                                                        >  1:211728/1‑282 (MQ=255)`  
                    `ggATGGGGTGATACCGCCGAGGACTTCTTGAATAACCCGTTCATAACCGAGCCTAAAACGTCTGTGCTTAAGTCCGGTAAGAACAAAGGCCAAGAGGTTACTAAATGGGTTAAACGCGACCCTGAGCCTCATGAGACGCTTTGGGACTGCATTAAGTCCATTGGCGCGAAGGCTGGTATGACCGAAGAGGATATTATCAAGCAGGGCCAAATGGCTCGAATCCTACGGTTCAACGAGTACAACTTTATTGACAAGGAGATTTACCTG‑TGGAGACCGTAACGTATATTGGTCTGGGTCTTtg                                                                                                                                                                                                                                                                                        >  1:371810/1‑301 (MQ=255)`  
                      `aTGGGGTGATACCGCCGAGGACTTCTTGAATAACCCGTTCATAACCGAGCCTAAAACGTCTGTGCTTAAGTCCGGTAAGAACAAAGGCCAAGAGGTTACTAAATGGGTTAAACGCGACCCTGAGCCTCATGAGACGCTTTGGGACTGCATTAAGTCCATTGGCGCGAAGGCTGGTATGACCGAAGAGGATATTATCAAGCAGGACCAAATGGCTCGAATCCTACGGTTCAACGAGTACAACTTTATTGACAAGGAGATTTACCTG‑TGGAGACCGTAACGTATATTGGTCTGGGTCTTtgtg                                                                                                                                                                                                                                                                                      <  1:435663/301‑1 (MQ=255)`  
                         `gggTGATACCGCCGAGGACTTCTTGAATAACCCGTTCATAACCGAGCCTAAAACGTCTGTGCTTAAGTCCGGTAAGAACAAAGGCCAAGAGGTTACTAAATGGGTTAAACGCGACCCTGAGCCTCATGAGCCGCTTTGGGACTTCATTAAGTCCATTGGCGCGAAGGCTGGTATGACCGAAGAGGATCTTATCAAGCAGGGCCAAATGGCTCGAATCCTAAGGTTCAACGAGTACAACTTTATTGACAAGGAGATTTACCTG‑TGGTGACCGTAACGTATATTGGCCTGGGTCTTTGTGttt                                                                                                                                                                                                                                                                                   >  1:60116/1‑300 (MQ=255)`  
                             `ggagatgtgtataagagacagAATAACCCGTTCATAACCGAGCCTAAAACGTCTGTGCTTAAGTCCGGTAAGAACAAAGGCCAAGAGGTTACTAAATGGGTTAAACGCGACCCTGAGCCTCATGAGACGCTTTGGGACTGCATTAAGTCCATTGGCGCGAAGGCTGGTATGACCGAAGAGGATATTATCAAGCAGGGCCAAATGGCTCGAATCCTACGGTTCAACGAGTACAACTTTATTGACAAGGAGATTTACCTG‑TGGAGACCGTAACGTATATTGGTCTGGGTCTTTGTGTTCTCgg                                                                                                                                                                                                                                                                               <  1:376376/281‑1 (MQ=255)`  
                                            `ttcttGAATAACCCGTTCATAACCGAGCCTAAAACGTCTGTGCTTAAGTCCGGTAAGAACAAAGGCCAAGAGGTTACTAAATGGGTTAAACGCGACCCTGAGCCTCATGAGACGCTTTGGGACTGCATTAAGTCCATTGGCGCGAAGGCTGGTATGACCGAAGAGGATATTATCAAGCAGGGCCAAATGGCTCGAATCCTACGGTTCAACGAGTACAACTTTATTGACAAGGAGATTTACCTG‑TGGAGACCGTAACGTATATTGGTCTGGGTCTTTGTGTTCTCGGAGTGTGCCTCATTTc                                                                                                                                                                                                                                                                >  1:478773/1‑301 (MQ=255)`  
                                                          `gTTCATAACCGAGCCTAAAACGTCTGTGCTTAAGTCCGGTAAGAACAAAGGCCAAGAGGTTACTAAATGGGTTAAACGCGACCCTGAGCCTCATGAGACGCTTTGGGACTGCATTAAGTCCATTGGCGCGAAGGCTGGTATGACCGAAGAGGATATTATCAAGCAGGGCCAAATGGCTCGAATCCTACGGTTCAACGAGTACAACTTTATTGACAAGGAGATTTACCTG‑TGGAGACCGTAACGTATATTGGTCTGGGTCTTTGTGTTATCGGAGTGTGCCTCATTTCGTGGGGCCTTTggg                                                                                                                                                                                                                                                  >  1:167120/1‑301 (MQ=255)`  
                                                            `tCATAACCGAGCCTAAAACGTCTGTGCTTAAGTCCGGTAAGAACAAAGGCCAAGAGGTTACTAAATGGGTTAAACGCGACCCTGAGCCTCATGAGACGCTTTGGGACTGCATTAAGTCCATTGGCGCGAAGGCTGGTATGACCGAATAGGATATTATCAAGCAGGGCCAAATGGCTCGAATCCTACGGTTCAACGAGTACAACTTTATTGACAAGGAGATTTACCTG‑TGGAGACCGTAACGTATATTGGTCTGGGTCTTTGTGTTCTCGGAGTGTGCCTCATTTCGTGGGGCCTTTGGGAc                                                                                                                                                                                                                                                >  1:266774/1‑301 (MQ=255)`  
                                                               `tAACCGAGCCTAAAACGTCTGTGCTTAAGTCCGGTAAGAACAAAGGCCAAGAGGTTACTAAATGGGTTAAACGCGACCCTGAGCCTCATGAGACGCTTTGGGACTGCATTAAGTCCATTGGCTCGAAGGCTGGTATGACCGAAGAGGATATTATCAAGCAGGGCCAAATGGCTCGAATCCTACGGTTCAACGTGTTCAACTTTATTAACAAGGAGATTTACCTG‑TGGAGACCGTAACGTATATTGGGCTGGGTTTTTGTTTTCTCGTGgtgttgtctcttacaaccatctccgcgcacacc                                                                                                                                                                                                                                             >  1:252318/1‑272 (MQ=255)`  
                                                                              `cGTCTGTGCTTAAGTCCGGTAAGAACAAAGGCCAAGAGGTTACTAAATGGGTTAAACGCGACCCTGAGCCTCATGAGACGCTTTGGGACTGCATTAAGTCCATTGGCGCGAAGGCTGGTATGACCGAAGAGGATATTATCAAGCAGGGCCAAATGGCTCGAATCCTACGGTTCAACGAGTACAACTTTATTGACAAGGAGATTTACCTG‑TGGAGACCGTAACGTATATTGGTCTGGGTCTTTGTGTTCTCGGAGTGTGCCTCATTTCGTGGGGCCTTTGGGACTTAGCCAGAATAATCAAg                                                                                                                                                                                                                              >  1:327191/1‑301 (MQ=255)`  
                                                                                 `cTGTGCTTAAGTCCGGTAAGAACAAAGGCCAAGAGGTTACTAAATGGGTTAAACGCGACCCTGAGCCTCATGAGACGCTTTGGGACTGCATTAAGTCCATTGGCGCGAAGGCTGGTATGACCGAAGAGGATATTATCAAGCAGGGCCAAATGGCTCGAATCCTACGGTTCAACGAGTACAACTTTATTGACAAGGAGATTTACCTG‑TGGAGACCGTAACGTATATTGGTCTGGGTCTTTGTGTTCTCGGAGTGTGCCTCATTTCGTGGGGCCTTTGGGACTTAGCCAGAATAATCAAGTCg                                                                                                                                                                                                                           <  1:69334/301‑1 (MQ=255)`  
                                                                                     `gCTTAAGTCCGGTAAGAACAAAGGCCAAGAGGTTACTAAATGGGTTAAACGCGACCCTGAGCCTCATGAGACGCTTTGGGACTGCATTAAGTCCATTGGCGCGAAGGCTGGTATGACCGAAGAGGATATTATCAAGCAGGGCCAAATGGCTCGAATCCTACGGTTCAACGAGTACAACTTTATTGACAAGGAGATTTACCTG‑TGGAGACCGTAACGTATATTGGTCTGGGTCTTTGTGTTCTCGGAGTGTGCCTCATTTCGTGGGGCCTTTGGGACTTAGCCAGAATAATCAAGTAGTTaa                                                                                                                                                                                                                       >  1:474343/1‑300 (MQ=255)`  
                                                                                      `cTTAAGTCCGGTAAGAACAAAGGCCAAGAGGTTACTAAATGGGTTAAACGCGACCCTGAGCCTCAGGAGACGCTTTGGGACGGCATTAAGTCCATTGGCGCGAAGGCTGGTATGACCGAAGAGGATATTATCAAGCAGGGCCAAATGCCTCGAATCCTACGGTTCAACGAGTACAACTTTATTGACAAGGAGATTTACCTG‑TGGAGACCGTAACGTATATTGGTCTGGGTCTTTGTGTTCTCGGAGTGTGCCTCATTTCGTGGGGCCTTTGGGACTTAGCCAGAATAATCAAGTCGTTaca                                                                                                                                                                                                                      <  1:304227/301‑1 (MQ=255)`  
                                                                                          `gtgtataagagaaagaagtctaaGAGGTGACTAAAGGGGTTAAACGCGACGCTGAGTCCCAGGAGATGCTGTGAGCTGGCATTAAGTCCAGTGGCGAGAAGGCTGGTATGACCGAAGAGGAGATTATCAAGCAGGGCCAAATGGCTCGAATCCTACGGTTCAACGAGTACAACTTTATTGACAAGGAGATTTACCTG‑TGGAGACCGTAACGTATATTGGTCTGTTTCTTTGTGTTCTCGGAGTGTGCCTCATTTCGTGGGGCCTTTGGGACTTAGCCAGAATAATCAAGTCGTTACACGac                                                                                                                                                                                                                  <  1:94822/280‑1 (MQ=255)`  
                                                                                          `aGTCCGGTAAGAACAAAGGCCAAGAGGTTACTAAATGGGTTAAACGCGACCCTGAGCCTCATGAGACGCTTTGGGACTGCATTAAGTCCATTGGCGCGAAGGCTGGTATGACCGAAGAGGATATTATCAAGCAGGGCCAAATGGCTCGAATCCTACGGTTCAACGAGTACAACTTTATTGACAAGGAGATTTACCTG‑TGGAGACCGTAACGTATATTGGTCTGGGTCTTTGTGTTCTCGGAGTGTGCCTCATTTCGTGGGGCCTTTGGGACTTAGCCAGAATAATCAAGTCGTTACACGac                                                                                                                                                                                                                  >  1:501877/1‑301 (MQ=255)`  
                                                                                            `tCCGGTAAGAACAAAGGCCAAGAGGTTACTAAATTGGTTAAACGCGACCCTGAGCCTCATGAGACGCTTTGGGACTGCATTAAGTCCATTGGCGCGAAGGCTGGTATAACCGAAGAGGATATTATCAAGCAGGGCCAAATGGCTCGAATCCTACGGTTCAACGAGTACAACTTTATTGACAAGGAGATTTACCTG‑TGGAGACCGTAACGTATATTGGTCTGGGTCTTTGTGTTCTCGGAGTGTGCCTCATTTCGTGGGGCCTTTGGGACTTAGCCAGAATAATCAAGTCGTTACACGacac                                                                                                                                                                                                                <  1:367972/301‑1 (MQ=255)`  
                                                                                            `tCCAGTAATAAAAAAGTCCAAAAGGTTACTAAATGGGTTAATCGCGACCCTGAACCTCATGGGACGCTTTGGGACTGCATTAATTCCATTTGCGCGAAGGCTGGTATGACCGAAGAGGATATTATCAAGCAGGGCCAAATGGCTCGAATCCTACGTTTCAACGAGTACCACTTTATTAACAAGGAGATTTACCTG‑TGGAGACCGTAACGTATATTGGTCTGGGTCTTTGTATTCTCGGAGTGTGCCTCATTTCGTGGGGCCTTTGGGACTTAGCCAGAATAATCAAGTCGTTACACGacac                                                                                                                                                                                                                <  1:360055/301‑1 (MQ=255)`  
                                                                                                `gTAAGAACAAAGGCAAAGAGGTTACTAAATGGGTTAAACGCGACCCTGAGCATCATGAGACGCTTTGGGACTGCAATAAGTAAATTGGCGCGAAAGCTGGTATGACCGAAGAGGATATTATCAAGCAGGGCCAAATGGCTCGAATCCTACGATGTAACGAGTACAACTTTATTGACAAGGAGATTTACCTG‑TGGAGACCGTAACGTATATGGGTCTGGGTCTTTGTGTTCTCGGAGTGTGCCTCATTTCGTGGGGCCTTTGGGACTTAGCCAGAATAATCAAGTCGTTACACGACACTAAg                                                                                                                                                                                                            <  1:161208/301‑1 (MQ=255)`  
                                                                                                 `tAAGAACAAAGGCCAAGAGGTTACTAAATGGGTTAAACGCGACCATGAGCCTCATGAGACGCTTTGGGACTGCATTAAGTCCATTGGCGCGAAGGCTGGTATGACCGAAGAGGATATTATCAAGCAGGGCCAAATGGCTCGAATCCTACGGTTCAACGAGTACAACTTTATTGACAAGGAGATTTACCTG‑TGGAGACCGTAACGTATATTGGTCTGGGTCTTTGTGTTCTCGGAGTGTGCCTCATTTCGTGGGGCCTTTGGGACTTAGCCAGAATAATCAAGTCGTTACACGACACTAAGt                                                                                                                                                                                                           <  1:215727/301‑1 (MQ=255)`  
                                                                                                 `aaggAACAAAGGCCAAGAGGTTACTAAATGGGTTAAACGCGACCCTGAGCCTCATGAGACGCTTTGGGACTGCATTAAGTCCATTGGCGCGAAGGCTGGTATGACCGAAGAGGATATTATCAAGCAGGGCCAAATGGCTCGAATCCTACGGTGCAACGAGTACAACTTTATTGAAAAGGAGATTTACCTG‑TGGAGACCGTAACGTATATTGGTCTGGGTCTTTGTGTTCTCGGAGTGTGACTCATTTCGTGGGGCCTTTGGGACTTAGCCAGAATAATCAAGTCGTTACACGACACTAAGt                                                                                                                                                                                                           <  1:77868/298‑1 (MQ=255)`  
                                                                                                        `agagacagagagGTTACTAAATGGGTTAAACGCGACCCTGAGCCTCATGAGACGCTTTGGGACTGCATTAAGTCCATTGGCGCGAAGGCTGGTATGACCGAAGAGGATATTATCAAGCAGGGCCAAATGGCTCGAATCCTACGGTTCAACGAGTACAACTTTATTGACAAGGAGATTTACCTG‑TGGAGACCGTAACGTATATTGGTCTGGGTCTTTGTGTTCTAGGAGTGTGCCTCATTTCGTGGGGCCTTTGGGACTTAGCCAGAATAATCAAGTCGTTACACGACACTAAGTGATAAAc                                                                                                                                                                                                    <  1:275912/293‑1 (MQ=255)`  
                                                                                                        `aaaGGCCAATAGGTTACTAAATTGGTTAAACGCGACCCTGAGCCTCATGAGACGCTTTGGGACTGCATTAAGTCCATTGGCGCGAAGGCTGGTATGACCGAAGAGGATATTATCAAGCAGGGCCAAATGGCTCGAATCCTACGGTTCAACGAGTACAACTTTATTGACAAGGAGATTTACCTG‑TGGAGACCGTAACGTATATTGGTCTGGGTCTTTGTGTTCTCGGAGTGTGCCTCATTTCGTGGGGCCTTTGGGACTTAGCCAGAATAATCAAGTAGTTACACGACACTAAGTGATAAAc                                                                                                                                                                                                    <  1:245340/301‑1 (MQ=255)`  
                                                                                                          `aGGCGAAGAGGTTACAAAAGGGGTTAAACGCGACCCTGAGCCTCATGAGACGCTTTGGGACTGCATTAAGTCCATTGGCGCGAAGGCTGGTATGACCGAAGAGGATATTATCAAGCAGGGCCAAATGGCTCGAATCCTACGGTTCAACGAGTACAACTTTATTGACAAGGAGATTTACCTG‑TGGAGACCGTAACGTATATTGGTCTGGGTCTTTGTGTTCTCGGAGTGTGCCTCATTTCGTGGGGCCTTTGGGACTTAGCCAGAATAATCAAGTCGTTACACGACACTAAGTGATAAACTc                                                                                                                                                                                                  <  1:100620/301‑1 (MQ=255)`  
                                                                                                           `ggCCAAGAGGTTACTAAATGGGTTAAACTCGACCCTGAGCCTCATGAGACGCTTTGGGACTGCATTAAGTCCATTGGCGCGAAGTCTGGTATGACCGAAGAGGATATTATCAAGCAGGGCCAAATGGCTCGAATCCTACGGTTCAACGAGTACAACTTTATTGACAAGGAGATTTACCTG‑TGGAGACCGTAACGTATATTGGTCTGGGGCTTTGTGTTCTCGGAGTGTGCCTCATTTCGTGGGGCCTTTGGGACTTAGCCAGAATAATCAAGTCGTTACACGACACTAAGTGATAAACTCa                                                                                                                                                                                                 >  1:498200/1‑301 (MQ=255)`  
                                                                                                                 `gagGTTACTAAATGGGTTAAACGCGACCCTGAGCCTCATGAGACGCTTTGGGACTGCATTAAGTCCATTGGCGCGAAGGCTGGTATGACCGAAGAGGATATTATCAAGCAGGGCCAAATGGCTCGAATCCTACGGTTCAACGAGTACAACTTTATTGACAAGGAGATTTACCTG‑TGGAGACCGTAACGTATATTGGTCTGGGTCTTTGTGTTCTCGGAGTGTGCCTCATTTCGTGGGGCCTTTGGGACTTAGCCAGAATAATCAAGTCGTTACACGACACTAAGTGATAAAcctgtctctt                                                                                                                                                                                           >  1:205207/1‑292 (MQ=255)`  
                                                                                                                 `gacagtACTAAATGGGTTAAACGCGACCCTGAGCCTCATGAGACGCTTTGGGACTGCATTAAGTCCATTGGCGCGAAGGCTGGTATGACCGAAGAGGATATTATCAAGCAGGGCCAAATGGCTCGAATCCTACGGTTCAACGAGTACAACTTTATTGACAAGGAGATTTACCTG‑TGGAGACCGTAACGTATATTGGTCTGGGTCTTTGTGTTCTCGGAGTGTGCCTCATTTCGTGGGGCCTTTGGGACTTAGCCAGAATAATCAAGTCGTTACACGACACTAAGTGATAAACTCAAGGTcc                                                                                                                                                                                           <  1:401483/296‑1 (MQ=255)`  
                                                                                                                    `gTTACTAAATGGGTTAAACGCGACCCTGAGCCTCATGAGACGCTTTGGGACTGCATTAAGTCCATTGGCGCGAAGGCTGGTATGACCGAAGAGGATATTATCAAGCAGGGCCAAATGGCTCGAATCCTACGGTTCAACGAGTACAACTTTATTGACAAGGAGATTTACCTG‑TGGAGACCGTAACGTATATTGGTCTGGGTCTTTGTGTTCTCGGAGTGTGCCTCATTTCGTGGGGCCTTTGGGACTTAGCCAGAATAATCAAGTCGTTACACGACACTAAGTGATAAACTCAAGGTACCTa                                                                                                                                                                                        >  1:183615/1‑301 (MQ=255)`  
                                                                                                                              `gggTTAAACGCGACCCTGAGCCTCATGAGACGCTTTGGGACTGCATTAAGTCCATTGGCGCGAAGGCTGGTATGACCGAAGAGGATATTATCAAGCAGGGCCAAATGGCTCGAATCCTACGGTTCAACGAGTACAACTTTATTGACAAGGAGATTTACCTG‑TGGAGACCGTAACGTATATTGGTCTGGGTCTTTGTGTTCTCAGAGTGTGCCTCATTTCGTGGGGCCTTTGGGACTTAGCCAGAATAATCAAGTCGTTACACGACACTAAGTGATAAACTCAAGGTCCCTAAATTAATACg                                                                                                                                                                              >  1:256249/1‑301 (MQ=255)`  
                                                                                                                               `ggTTAAACGCGACCCTGAGCCTCATGAGACGCTTTGGGACTGCATTAAGTCCATTGGCGCGAAGGCTGGTATGACCGAATAGGATATTATCAAGCAGGGCCAAATGGCTCGAATCCTACGGTTCAACGAGTACAACTTTATTGACAAGGAGATTTACCTG‑TGGATACCGTAACGTATATTGGTCTGGGTCTTTGTGTTCTCGGAGTGTGCCTCATTTCGTGGGGCCTTTGGGACTTAGCCAGAATAATCAAGTCGTTACACGACACTAAGTGATAAACTCAAGGTCCCTAAATTAATActt                                                                                                                                                                             >  1:508048/1‑299 (MQ=255)`  
                                                                                                                               `ggTTAAACGCGACCCTGAGCCTCATGAGACGCTTTGGGACTGCATTAAGTCCATTGGCGCGAAGGCTGGTATGACCGAAGAGGATATTATCAAGCAGGGCCAAATGGCTCGAATCCTACGGTTCAACGAGTACAACTTTATTGACAAGGAGATTTACCTG‑TGGAGACCGTAACGTATATTGGTCTGGGTCTTTGTGTTCTCGGAGTGTGCCTCATTTCGTGGGGCCTTTGGGACTTAGCCAGAATAATCAAGTCGTTACACGACACTAAGTGATAAACTCAAGGTCCCTAAATTAATACGa                                                                                                                                                                             >  1:430024/1‑301 (MQ=255)`  
                                                                                                                               `ggTTAAACGCGACCCTGAGCCTCATGAGACGCTTTGGGACTGCATTAAGTCCATTGGCGCGAAGGCTGGTATGACCGAAGAGGATATTATCAAGCAGGGCCAAATGGCTCGAATCCTACGGTTCAACGAGTACAACTTTATTGACAAGGAGATTTACCTG‑TGGAGACCGTAACGTATATTGGTCTGGGTCTTAGTGTTCTCGGAGTGTGCCTCATTTCGTGGGGCCTTTGGGACTTAGCCATAATAATCAAGTCGTTACACGACACTAAGTGATAAACTTCAGGTCCCTAAATTACTACGa                                                                                                                                                                             >  1:73938/1‑301 (MQ=255)`  
                                                                                                                                       `gcgACGCTGAGCCTCATGAGACGCTGTGGGACTGCATTAAGTCCATTGGCGCGAAGGCTGGTATGACCGAAGAGGATATTATCAAGCAGGGCCAAATGGCTCGAATCCTACGGTTCAACGAGTACAACTTTATTGACAAGGAGATTTACATG‑TGGAGACCGTAACGTAGATTGGTCTGGGTCTTTGTGTTCTCGGAGTGTGCCTCATTTCGTGGGGCCTTTGGGACTTAGCCAGAATAATCAAGTCGTTACACGACACTAAGTGATAAACTCAAGGTCCCTAAATTAATACGACTCACtat                                                                                                                                                                     <  1:397643/301‑1 (MQ=255)`  
                                                                                                                                          `aCCCTGAGCCTTATGAGACGCTTTGGGACTGCATTAAGTCCATTGGCGCGAAGGCTGGTATGACCGAAGAGGATATGATCAAGCAGGGCCAAATGGCTCGAATCCTACGGTTCAACGAGTACAACTTTATTGACAAGGAGATTTACCTG‑TGGAGACCGTAACGTATATTGGTCTGGGTCTTTGTGTTCTCGGAGTGTGCCTCATTTCGTGGGGCCTTTGGGACTTAGCCAGAATAATAAAGTCGTTACACGACACTAAGTGATAAACTCAAGGTCCCTAAATTAATACGACTCACTATAgg                                                                                                                                                                  <  1:406390/301‑1 (MQ=255)`  
                                                                                                                                          `aCCCTGAGCCTCATGAGACGCTTTGGTACTGCATTAAGTCCATTGGCGCGAAGGCTGGTATGACCGAAGATGATATTATCAAGCAGGGCCAAATGGCTCGAATCCTATGGTTCAACGAGTACAACTTTATTGACAAGGAGATTTACCTG‑TGGAGACCGTAACGTATATTGGTCTGGGTCTTTGTGTTCTCGGAGTGTGCCTCATTTCGTGGGGCCTTTGGGACTTAGCCAGAATAATCAAGTCGTTACACGACACTAAGTGATAAACTCAAGGTCCCTAAATTAATACGACTCACTATAgg                                                                                                                                                                  >  1:412018/1‑301 (MQ=255)`  
                                                                                                                                          `aCCCTGAGCCTCATGAGACGCTTTGGGACTGCATTAAGTCCATTGTCGCGAAGGCTGGTATGACCGAAGAGGATATTATCAAGCAGGGCCAAATGGCTCGAATCCTACGGTTCAACGAGTACAACTTTATTGACAAGGAGATTTACCTG‑TGGAGACCGTAACGTATATTGGTCTGGGTCTTTGTGTTCTCGGAGTGTGCCTCATTTCGTGGGGCCTTTGGGACTTAGCCAGAATAATCAAGTCGTTACACGACACTAAGTGATAAACTCAAGGTCCCTAAATTAATACGACTCACTATAgg                                                                                                                                                                  >  1:105123/1‑301 (MQ=255)`  
                                                                                                                                          `aCCCTGAGCCTCATGAGACGCTTTGGGACTGCATTAAGTCCATTGGCTCGAAGGCTGGTATGACCGAAGAGGATATTATCAAGCAGGGCCAAATGGCTCGAATCCTACGGTTCACCGAGTACAACTTTATTGACAAGGAGATTTACCTG‑TGGAGACCGTAGCGTATATTGGTCTGGGTCTTTGTGTTCTCGGAGTGTGCCTCATTTCGTGGGGCCTTTGGGACTTCGCCAGAATAATCAAGTCGTTACACGACACTAAGTGATAAACTCAAGGTCCCTAAATTAATCCGACTCACTATAgg                                                                                                                                                                  >  1:279441/1‑301 (MQ=255)`  
                                                                                                                                          `aCCCTGAGCCTCATGAGACGCTTTGGGACTGCATTAAGTCCATTGGCGCGAAGGCTGGTATGACCGAAGAGGATATTATCAAGCAGGGCCAAATGGCTCGAATCCTACGGTTCAACGAGTACAACTTTATTGACAAGGAGATTTACCTG‑TGGAGACCGTAACGTATATTGGTCTGGGTCTTTGTGTTCTCGGAGTGTGCCTCATTTCGTGGGGCCTTTGGGACTTAGCCAGAATAATCAAGTCGTTACACGACACTAAGTGATAAACTCAAGGTCCCTAAATTAATACGACTCCCTATAgg                                                                                                                                                                  >  1:346536/1‑301 (MQ=255)`  
                                                                                                                                          `aCCCTGAGCCTCATGAGACGCTTTGGGACTGCATTAAGTCCATTGGCGCGAAGGCTGGTATGACCGAAGAGGATATTATCAAGCAGGGCCAAATGGCTCGAATCCTACGGTTCAACGAGTACAACTTTATTGACAAGGAGATTTACCTG‑TGGAGACCGTAACGTATATTGGTCTGGGTCTTTGTGTTCTCGGAGTGTGCCTCATTTCGTGGGGCCTTTGGGACTTAGCCAGAATAATCAAGTCGTTACACGACACTAAGTGATAAACTCAAGGTCCCTAAATTAATACGACTCACTATAgg                                                                                                                                                                  >  1:351404/1‑301 (MQ=255)`  
                                                                                                                                          `aCCCTGAGCCTCATGAGACGCTTTGGGACTGCATTAAGTCCATTGGCGCGAAGGCTGGTATGACCGAAGAGGATATTATCAAGCAGGGCCAAATGGCTCGAATCCTACGGTTCAACGAGTACAACTTTATTGACAAGGAGATTTACCTG‑TGGAGACCGTAACGTATATTGGTCTGGGTCTTTGTGTTCTCGGAGTGTGCCTCATTTCGTGGGGCCTTTGGGACTTAGCCAGAATAATCAAGTCGTTACACGACACTAAGTGATAAACTCAAGGTCCCTAAATTAATACGACTCACTATAgc                                                                                                                                                                  >  1:199078/1‑300 (MQ=255)`  
                                                                                                                                           `cccTGAGCCTCATGAGACGCTTTGGGACTGCATTAAGTCCATTGGCGCGAAGGCTGGTATGACCGAAGAGGATATTATCAAGCAGGGCCAAATGGCTCGAATCCTACGGTTCAACGAGTACAACTTTATTGACAAGGAGATTTACCTG‑TGGAGACCGTAACGTATATTGGTCTGGGTCTTTGTGTTCTCGGAGTGTGCCTCATTTCGTGGGGCCTTTGGGACTTAGCCAGAATAATCAAGTCGTTACACGACACTAAGTGATAAACTCAAGGTCCCTAAATTAATACGACTCACTATAggg                                                                                                                                                                 <  1:335991/301‑1 (MQ=255)`  
                                                                                                                                               `gAGCCTCATGAGACGCTTTGGGACTGCATTAAGTCCATTGGCGCGAAGGCTGGTATGACCGAAGAGGATATTATCAAGCAGGGCCAAATGGCTCGAATCCTACGGTTCAACGAGTACAACTTTATTGACAAGGAGATTTACCTG‑TGGAGACCGTAACGTATATTGGTCTGGGTCTTTGTGTTCTCGGAGTGTGCCTCATTTCGTGGGGCCTTTGGGACTTAGCCAGAATAATCAAGTCGTTACACGACACTAAGTGATAAACTCAAGGTCCCTAAATTAATACGACTCACTATAGGGAGAt                                                                                                                                                             >  1:261845/1‑301 (MQ=255)`  
                                                                                                                                                             `gCTTTGGGACTGCATTAAGTCCATTGGCGCGAAGGCTGGTATGACCGAAGAGGATATTATCAAGCAGGGCCAAATGGCTCGAATCCTACGGTTCAACGAGTACAACTTTATTGACAAGGAGATTTACCTG‑TGGAGACCGTAACGTATATTGGTCTGGGTCTTTGTGTTCTCGGAGTGTGCCTCATTTCGTGGGGCCTTTGGGACTTAGCCAGAATAATCAAGTCGTTACACGACACTAAGTGATAAACTCAAGGTCCCTAAATTAATACGACTCACTATAGGGAGATAGGGGCCTTTACGa                                                                                                                                               >  1:283551/1‑301 (MQ=255)`  
                                                                                                                                                              `cTTTGGGACTGCATTAAGTCCATTGGCGCGAAGGCTGGTATGACCGAAGAGGATATTATCAAGCAGGGCCAAATGGCTCGAATCCTACGGTTCAACGAGTACAACTTTATTGACAAGGAGATTTACCTG‑TGGAGACCGTAACGTATATTGGTCTGGGTCTTTGTGTTCTCGGAGTGTGCCTCATTTCGTGGGGCCTTTGGGACTTAGCCAGAATAATCAAGTCGTTACACGACACTAAGAGATAAACTCAAGGTCCCTAAATTAATACGACTCACGTTAGGgactgtctcttatacacatt                                                                                                                                              >  1:495208/1‑283 (MQ=255)`  
                                                                                                                                                                        `gCATTAAGTCCATTGGCGCGAAGGCTGGTATGACCGAAGAGGATATTATCAAGCAGGGCCAAATGGCTCGAATCCTACAGTTCAACGAGTACAACTTTATTGACAAGGAGATTTACCTG‑TGGAGACCGTAACGTATATTGGTCTGGGTCTTTGTGTTCTCGGAGTGTGCCTCATTTCGTGGGGCCTTTGGGACTTAGCCAGAATAATCAAGTAGTTACACGACACTAAGTGATAAACTCAAGGTCCCTAAATTAATAAGACTCACTATAGGGAGATAGGGGCCTTTACGATTATTAATTTa                                                                                                                                    >  1:86268/1‑301 (MQ=255)`  
                                                                                                                                                                         `cATTAAGTCCATTGGCGCGAAGGCTGGTATGACCGAAGAGGATATTATCAAGCAGGGCCAAATGGCTCGAATCCTACGGTTCAACGAGTACAACTTTATTGACAAGGAGATTTACCTG‑TGGAGACCGTAACGTATATTGGTCTGGGTCTTTGTGTTCTCGGAGTGTGCCTCATTTCGTGGGGCCTTTGGGACTTAGCCAGAATAATCAAGTCGTTACACGACACTAAGTGATAAACTCAAGGTCCCTAAATTAATACGACTCACTATAgggctgtctcttatacacatctccgagcccacg                                                                                                                                   >  1:193207/1‑271 (MQ=255)`  
                                                                                                                                                                                         `gcgAAGGCTGGTATGACCGAAGAGGATATTATCAAGCAGGGCCAAATGGCTCGAATCCTACGGTTCAACGAGTACAACTTTATTGACAAGGAGATTTACCTG‑TGGAGACCGTAACGTATATTGGTCTGGGTCTTTGTGTTCTCGGAGTGTGCCTCATTTCGTGGGGCCTTTGTGACTTAGCCATAATAATCAAGTCGTTACACGACACTAAGTGATAAACTCAAGGTCCCTAAATTAATACGACTCACTATAGGGAGATAGGGGCCTTTACGATTATTACTTTAAGATTTAACTCTAagag                                                                                                                   <  1:136721/301‑1 (MQ=255)`  
                                                                                                                                                                                              `ggCTGGTATGACCGAAGAGGATATTATCAAGCAGGGCCAAATGGCTCGAATCCTACGGTTCAACGAGTACAACTTTATTGACAAGGAGATTTACCTG‑TGGAGACCGTAACGTATATTGGTCTGGGTCTTTGTGTTCTCGGAGTGTGCCTCATTTCGTGGGGCCTTTGGGACTTAGCCAGAATAATCAAGTCGTTACACGACACTAAGTGATAAACTCAAGGTCCCTAAATTAATACGACTCACTATAGGGAGATAGGGGCCTTTACGATTATTACTTTAAGATTTAACTCTAAGAGGAAtt                                                                                                              >  1:291349/1‑300 (MQ=255)`  
                                                                                                                                                                                                 `ttgTATGACCGAAGAGGATATTATCAAGCAGGGCCAAATAGCTCGAATCCTACGGTTCAACGAGTACAACTTCATTGACAAGGAGATTTACCTG‑TAGAGACCGTAACGTATATTGGTCTGGGTCTTTGTGTTCTCGGAGTGTGCCTCATTTCGTGGTGCCTTTGGGACTTAGCCAGAATAATCAAGTCGTTACACGACACTAAGTGATAAACTCAAGGTCCCTAAATTACTACGACTCACTATAGGGAGATAGGGGCCTTTACGATTATTACTTTAAGATTTAACTCTAAGAGGAATCttt                                                                                                           <  1:124411/299‑1 (MQ=255)`  
                                                                                                                                                                                                  `ggTATTACCGAAGAGGATATTATCAAGCAGGGCCAAATGGCTCGAATCCTACGGTTCAACGAGTACAACTTTATTGACAAGGAGATTTACCTG‑TGGAGACCGTAACGTATATTGGTCTGGGTCTTTGTGTTCTCGGAGTGTGCCTCATTTCGTGGGGCCTTTGGGACTTAGCCAGAATAATCAAGTCGTTACACGACACTAAGTGATAAACTCAAGGTCCCTAAATTAATACGACTCACTATAGGGAGATAGGGGCCTTTACGATTATTACttctgtctcttatacacatctccgagcccc                                                                                                          >  1:38038/1‑273 (MQ=255)`  
                                                                                                                                                                                                     `aTGACCGAAGAGGTGCTAAGCAAGGAGGGGCAAATGGCTCGAATCAGACGGTTGAACGAGTAAAACATTAATGACAAGGATATTTACCTG‑TGGAGACCGTAACGTATAGTGGTCTGGGTCTATGTGTTCTCGGAGTGTGCCTCATTTCATGGGGCCTTTGGGAGTTAGCCAGAATAATCAAGTCGTTACACGACACTAAGTGAGAAACTCAAGGTCCCTAAATTAATACGACTCACGATATGGAGATAGGTGCCTTTACGATTATTACTTTAAGATTTAACTCTAAGAGGAATCTttatta                                                                                                       <  1:416971/301‑1 (MQ=255)`  
                                                                                                                                                                                                        `agacagaagGATATTATCAAGCAGGGCCAAATGGCTCGAATCCTACGGTTCAACGAGTACAACTTTATTGACAAGGAGATTTACCTG‑TGGAGACCGTAACGTGTATTGGTCTGGGTCTTTGTGTTCTCGGAGTGTGCCTCATTTCGTGGGGCCTTTGGGACTTAGCCAGAATAATCAAGTCGTTACACGACACTAAGTGATAAACTCAAGGTCCCTAAATTAATACGACTCACTATAGGGAGATAGGGGCCTTTACGATTATTACTTTAAGATTTAACTCTAAGAGGAATCTTTATTATGt                                                                                                    <  1:389093/294‑1 (MQ=255)`  
                                                                                                                                                                                                        `aCCGAAGAGGATATTATCAAGCAGGGCCAAATTGCTCGAATCCTACGGTTCAACGAGTACAACTTTATTGACAAGGAGATTTACCTG‑TGGAGACCGTAACGTATATTGGTCTGGGTCTTTGGGTTCTCGGAGTGTGCCTCATTTCGTTGGGCCTTTGGGACTTAGCCAGAATAATCAAGTCGTTACACGACACTAAGTGATAAACTCAAGGTCCCTAAATTAATACGACTCACTATAGGGAGATAGGGGCCTTTACGATTATTACTTTAAGATTTAACTCTAAGAGGAATCTTTATTATGt                                                                                                    <  1:172612/301‑1 (MQ=255)`  
                                                                                                                                                                                                        `aCCGAAGAGGATATTATCAAGCAGGGCCAAATGGCTCGAATCCTACGGTTCAACGAGTACAACTTTATTGACAAGGAGATTTACCTG‑TGGAGACCGTAACGTATATTGGTCTGGGTCTTTGTGTTCTCGGAGTGTGCCTCATTTCGTGGGGCCTTTGGGACTTAGCCAGAATAATCAAGTCGTTACACTACACTAAGTGATAAACTCAAGGTCCCTAAATTAATACGACTCACTATAGGGAGATAGGGGCCTTTACGATTATTACTTTAAGATTTAACTCTAAGAGGAATCTTTATTATGt                                                                                                    >  1:248726/1‑301 (MQ=255)`  
                                                                                                                                                                                                                `ggATATTATCAAGCAGGGCCAAATGGCTCGAATCCTACGGTTCAACGAGTACAACTTTATTGACAAGTAGATTTACCTG‑TGGAGACCGTAACGTATATTGGTCTGGGTCTTTGTGTTCTCGTAGTGTGCCTCATTTCGTGGGGCCTTTGGGACTTAGCCAGAATAATCAAGTCGTTACACGACACTAAGTGATAAACTCAAGGTCCCTAAATTAATACGACTCACTATAGGGAGATAGGGGCCTTTACGATTATTACTTTAAGATTTAACTCTAAGAGGAATCTTTATTATGTTAACACCt                                                                                            >  1:469196/1‑301 (MQ=255)`  
                                                                                                                                                                                                                `ggATATTATCAAGCAGGGCCAAATGGCTCGAATCCTACGGTTCAACGAGTACAACTTTATTGACAAGGAGATTTACCTG‑TGGAGACCGTAACGTATATTGGTCTGGGTCTTTGTGTTCTCGGAGTGTGCCTCATTTCGTGGGGCCTTTGGGACTTAGCCAGAATAATCAAGTCGTTACACGACACTAAGTGATAAACTCAAGGTCCCTAAATTAATACGACTCACTATAGGGAGATAGGGGCCTTTACGATTATTACTTTAAGATTTAACTCTAATAGGAATCTTTATTATGTTAACACCt                                                                                            >  1:271252/1‑301 (MQ=255)`  
                                                                                                                                                                                                                `ggATATTATCAAGCAGGGCCAAATGGCTCGAATCCTACGGTTCAACGAGTACAACTTTATTGACAAGGAGATTTACCTG‑TGGAGACCGTAACGTATATTGGTCTGGGTCTTTGTGTTCTCGGAGTGTGCCTCATTTCATGGGGCCTTTGGGACTTAGCCAGAATAATCAAGTCGTTACACGACACTAAGTGATAAACTCAAGGTCCCTAAATTAATACGACTCACTATAGGGAGATAGGGGCCTTTACGATTATTACTTTAAGATTTAACTCTAAGAGGAATCTTTATTATGTTAACACCt                                                                                            >  1:236919/1‑301 (MQ=255)`  
                                                                                                                                                                                                                   `tattatCAAGCAGGGCCAAATGGCTCGAATCCTACGGTTCAACGAGTACAACTTTATTGACAAGGAGATTTACCTG‑TGGAGACCGTAACGTATATTGGTCTGGGTCTTTGTGTTCTCGGAGTGTGCCTCATTTCGTGGGGCCTTTGGGACTTAGCCAGAATAATCAAGTCGTTACACGACACTAAGTGATAAACTCAAGGTCCCTAAATTAATACGACTCACTATAGGGAGATAGGGGCCTTTACGCTTATTACTTTAAGATTTAACTCTAAGAGGAATCTTTATTATGTTAACAccctgt                                                                                         >  1:286550/1‑297 (MQ=255)`  
                                                                                                                                                                                                                    `gttatCAAGCAGGACCAAATGGCTCGAATCCTACGGTTCAACGAGTACAACTTTATTGACAAGGAGATTTACCTG‑TGGAGACCGTAACGTATATTGGTCTGGGTCTTTGTGTTCTCGGAGTGTGCCTCATTTCGTGGGGCCTTTGGGACTTAGCCAGAATAATCAAGTCGTTACACGACACTAAGTGATAAACTCAAGGTCCCTAAATTAATACGACTCACTATAGGGAGATAGGGGCCTTTACGATTATTACTTTAAGATTTAACTCTAAGAGGAATCTTTATTATGTTAACACCTATTa                                                                                        <  1:14526/300‑1 (MQ=255)`  
                                                                                                                                                                                                                     `ttatCAAGCAGGGCCAAATGGCTCGAATCCTACGGTTCAACGCGTACAACTTTATTGACAAGGAGATTTACCTG‑TGGAGCCCGTAACGTATATTGGTCTGGGGCTTTGTGTTCTCGGAGTGTGCCTCATTTCGTGGGGCCTTTGGGACTTAGCCAGAATAATCAAGTCGTTCCTCGACACTAAGTGATACACTCAAGCTCACTATATTAATACGACTCACTCTAGCGCGATATGCGCCTTTACGATTATTACTTTAAGACTTAACTCTAAGAGGCATCTTTCTTATTTTAACACCTATTaa                                                                                       >  1:451127/1‑301 (MQ=255)`  
                                                                                                                                                                                                                     `ttatCAAGCAGGGCCAAATGGCTCGAATCCTACGGTTCAACGAGTACAACTTTATTGACAAGGAGATTTACCTG‑TGGAGACCGTAACGTATATTGGTCTGGGTCTTTGTGTTCTCGGAGTGTGCCTCATTTCGTGGGGCCTTTGGGACTTAGCCAGAATAATCAAGTCGTTACACGACACTAAGTGATAAACTCAAGGTCCCTAAATTAATACTACTCACTATAGGGAGATAGGGGCCTTTACGATTATTACTTTAAGATTTAACTCTAAGATGAATCTTTATTATGTTAACACCtgtctc                                                                                       >  1:462811/1‑296 (MQ=255)`  
                                                                                                                                                                                                                     `ttatCAAGCAGGGCCAAATGGCTCGAATCCTACGGTTCAACGAGTACAACTTTATTGACAAGGAGATTTACCTG‑TGGAGACCGTAACGTATATTGGTCTGGGTCTTTGTGTTCTCGGAGTGTGCCTCATTTCGTGGGGCCTTTGGGACTTAGCCAGAATAATCAAGTCGTTACACGACACTAAGTGATAAACTCAAGGTCCCTAAATTAATACGACTCACTATAGGGAGATAGGGGCCTTTACGATTATTACTTTAAGATTTAACTCTAAGAGGAATCTTTATTATGTTAACACCtgtcct                                                                                       >  1:51767/1‑296 (MQ=255)`  
                                                                                                                                                                                                                     `ttatCAAGCAGGGCAAAATGGCTCGAATCCTACGGTTCAACGAGTACAACTTTATTGACAAGGAGATTTACCTG‑TGGAGACCGTAACGTATATTGGTCTGGGTCTTTGTGTTCTCGGAGTGTGCCTGATTTCGTGGGGCCTTTGGGACTTAGCCAGAATAATCAAGTCGTTACAAGACACTAAGTGATAAACTCAAGGTACCTAAATTAATACGAATACCTATAGGGAGATAGGGGACTTTACGATTATTACTTTAAGATTTAACTCTAAGAGGAATATTTATTATGTTAACACCTATTaa                                                                                       >  1:309670/1‑301 (MQ=255)`  
                                                                                                                                                                                                                                     `atgtttataagagacagggTTCAACGAGTACAACTTTATTGACAAGGAGATTTACCTG‑TGGAGACCGTAACGTATATTGGTCTGGGTCTTTGTGTTCTCGGAGTGTGCCTCATTTCGTGGGGCCTTTGGGACTTAGCCAGAATAATCAAGTCGTTACACGACACTAAGTGATAAACTCAAGGTCCCTAAATTAATACGACTCACTATAGGGAGATAGGGGCCTTTACGATTATTACTTTAAGATTTAACTCTAAGAGGAATCTTTATTATGTTAACACCTATTAACCAATTACTTAAGAAc                                                                       <  1:225544/284‑1 (MQ=255)`  
                                                                                                                                                                                                                                            `cGAATCCTACGGTTCAACGAGTACAACTTTATTGACAAGGAGATTTACCTG‑TGGAGACCGTAACGTATATTGGTCTGGGTCTTGGTGTGCTCGGAGTGTGCCTCATTTCGTGGGGCCTTTGGGACTTAGCCAGAATAATCAAGTCGTTACACGACACTAAGTGATAAACTCAAGGTCCCTAAATTAATACGACTCACTATAGGGAGATAGGGGCCTTTACGATTATTACTTTAAGATTTAACTCTAAGAGGAATCTTTATTATGTTAACACCTATTAACCAATTACTTAAGAACCCTAACg                                                                <  1:355292/301‑1 (MQ=255)`  
                                                                                                                                                                                                                                            `cGAATCCTACGGTTCAACGAGTACAACTTTAGTGACAAGGAGATGTACCTG‑TGGAGACCGTAACGTATATTGGTCTGGGTCTTTGTGTTCTCGGAGTGTGCCTCATTTCGTGGGGCCTTTGGGACTTAGCCAGAATAATCAAGTCGTTACACGACACTAAGTGATAAACTCAAGGTCCCTAAATTAATACGACTCACTATAGGGAGATAGGGGCCTTTACGATTATTACTTTAAGATTTAACTCTAAGAGGAATCTTTATTATGTTAACACCTATTAACCAATTACTTAAGAACCCTAACg                                                                <  1:425942/301‑1 (MQ=255)`  
                                                                                                                                                                                                                                                  `caacgcttcaagaggaaaaccTTAATTGACAA‑GAGGTTTACCTGTTGAAGACGTAAACTTATATTGGT‑TGTGTTCTTGTGTTCTCGGAGTGTGCCTCATTTCGTGGGGCCTTTGGGACTTAGCCAGAATAATCAAGTCGTTACACGACACTAAGTGATAAACTCAAGGTCCATAAATTAATACGACTCACTATAGGGAGATAGGGGCCTTTACGATTATTACTTTAAGATTTAACTCTAAGAGGAATCTTTATTATGTTAACACCTATTAACCAATTACTTAAGAACCCTAACGATATTcc                                                         <  1:300574/281‑1 (MQ=255)`  
                                                                                                                                                                                                                                                  `cTACGGTTCAACGAGTACAACTTTATTGACAAGGAGATTTACCTG‑TGGAGACCGTAACGTATATTGGTCTGGGTCTTTGTGTTCTCGGAGTGTGCCTCATTTCGTGGGGCCTTTGGGACTTAGCCAGAATAATCAAGTCGTTACACGACACTAAGTGATAAACTCAAGGTCCCTAAATTAATATGACTCACTATAGGGAGATAGGGGCCTTTACGATTATTACTTTAAGATTTAACTCTAAGAGGAATCTTTATTATGTTAACACCTATTAACCAATTACTTAATAACCATAACGATATTc                                                          >  1:177983/1‑301 (MQ=255)`  
                                                                                                                                                                                                                                                   `aagagacagaagAGTACAACTTTATTGACAAGGAGATTTACCTG‑TGGAGACCGGAACGTATATTGGTCTGGGTCTTTGTGTTCTCGGAGTGTGCCTCATTTCGTGGGGCCTTTGGGACTTAGCCAGAATAATCAAGTCGTTACACGATACTAAGTGATAAACTCAAGGTCCCTAAATGAATACGACTCACTATAGGGAGATAGGGGCCTTTACGATTATTACTTTAAGATTTAACTCTAAGAGGAATCTTTATTATGTTAACACCTATTAACCAATTACTTAAGAACCCTAACGATATTcc                                                         <  1:486365/290‑1 (MQ=255)`  
                                                                                                                                                                                                                                                      `ggTTCAACGAGTACAACTTTATTGACAAGGAGATTTACCTG‑TGGAGACCGTAACGTATATTGGTCTGGGTCTTTGTGTTCTCGGAGTGTGCCTCATTTCGTGGGGCCTTTGGGACTTAGCCAGAATAATCAAGTCGTTACACGACACTAAGTGATAAACTCAAGGTCCCTAAATTAATACGACTCACTATAGGGAGATAGGGGCCTTTACGATTATTACTTTAAGCTTCAACTCTAAGAGGAATCTTTATTATGTTAACACCTATTAACAAATCAcctgtctcttatacacatctcctatc                                                      >  1:55120/1‑276 (MQ=255)`  
                                                                                                                                                                                                                                                        `ttCAACGAGTAGAACTGTATTGAGAAGGAGATTTAACTG‑TGGAGACCGTAACGTAGAATGGGCGGGGTATTTGTGTTCTGGGGGTGTGCCTCATTTCGTGGGGCCGTTGGGACTTAGCCAGAATAATCAAGTCGTTACACGACACTAAGTGATAAACTCAAGGTCCCTAAATTAATACGACTCACTATAGGGAGATAGGGGCCTTTACGATTATTACTTTAAGATTTAACTCTAAGAGGAATCTTTATTATGTTAACACCTATTAACCAATTACTTAAGAACCCTAACGATATTCCAGATg                                                    <  1:318622/301‑1 (MQ=255)`  
                                                                                                                                                                                                                                                             `ggagatgtgtataagagACAGGGAGATTTACCTG‑TGGAGACCGTAACGTATATTGGTCTGGGTCTTTGTGTTCTCGGAGTGTGCCTCATTTCGTGGGGCCTTTGGGACTTAGCCAGAATAATCAAGTCGTTACACGACACTAAGTGATAAACTCAAGGTCCCTAAATTAATACGACTCACTATAGGGAGATAGGGGCCTTTACGATTATTACTTTAAGATTTAACTCTAAGAGGAATCTTTATTATGTTAACACCTATTAACCAATTACTTAAGAACCCTAACGATATTCCAGATGTACCt                                               <  1:446747/285‑1 (MQ=255)`  
                                                                                                                                                                                                                                                             `cGAGTACAACTTTATTGACAAGGAGATTTACCTG‑TGGAGACCGTAACGTATATTGGTCTGGGTCTTTGTGTTCTCGGAGTGTGCCTCATTTCGTGGGGCCTTTGGGACTTAGCCAGAATAATCAAGTCGTTACACGACACTAAGTGATAAACTCAAGGTCCCTAAATTAATACGACTCACTATAGGGAGATAGGGGCCTTTACGATTATTACTTTAAGATTTAACTCTAAGAGGAATCTTTATTATGTTAACACCTATTAACCAATTACTTAAGAACCCTAACGATATTCCAGATGTACCt                                               <  1:385011/301‑1 (MQ=255)`  
                                                                                                                                                                                                                                                                     `aCTTTAGTGACAAGGAGATTTACCGG‑TGGAGACCGTAACGTATATTGGTCTGGGTCTTTGTTTTCTCGGAGTGTGCCTCATTTCGTGGGGCCTTTGGGACTTAGCCAGAATAATCAAGTCGTTACACGACACTAAGTGATAAACTCAAGGTCCCTAAATTAATACGACTAACTATAGGGAGATAGGGGCCTTTACGATTATTACTTTAAGATTTAACTCTAAGAGGAATCTTTATTATGTTAACACCTATTAACCAATTACTTAAGAACCCTAACGATATTCCAGATGTACCTCGTGCAAc                                       <  1:140868/301‑1 (MQ=255)`  
                                                                                                                                                                                                                                                                      `cTTTATTTACAAGGAGATTTACCTG‑TGGAGACCGTAACGTATATTGGTCTGGGTCTTTGTGTTCTCGGAGTGTGCCTCATTTCGTGGGGCCTTTGGGACTTAGCCAGAATAATCAAGTCGTTACACGACACTAAGTGATAAACTCAAGGTCCCTAAATTAATACGACTCACTATAGGGAGATAGGGGCCTTTACGATTATTACTTTAAGATTTAACTCTAAGAGGAATCTTTATTATGTTAACACCTATTAACCAATTACTTAAGAACCCTAACGATATTCCAGATGTACCTCGTGCAAcc                                      >  1:364003/1‑301 (MQ=255)`  
                                                                                                                                                                                                                                                                        `ttATTGACAAGGAGATTTACCTG‑TGGAGACCGTAACGTATAGTGGTCTGGGTCTTTGTGTTCTCGGAGTGTGCCTCATTTCGTGGGGCCTTTGGGACTTAGCAAGAATAATCAAGTCGTTACACGACACTAAGTGATAAACTCAAGGTCCCTAAATTAATACGACTCACTATAGGGAGATAGGGGCCTTTACGATTATTACTTTAAGATTTAACTCTAAGAGGAATCTTTATTATGTTAACACCTATTAACCAATTACTTAAGAACCCTAACGATATTCCAGATGTACCTCGTGCAACCGc                                    <  1:90150/301‑1 (MQ=255)`  
                                                                                                                                                                                                                                                                                  `ggAGATTTACCTG‑TGGAGACCGTAACGTATATTGGTCTGGGTCTTTGTGTTCTCTGAGTGTGCCTCATTTCGTGGGGCCTTTGGGCCTTAGCCATAATAATCAAGTCGTTACACGACACTAAGTTATAAACTCAAGGTCCCTAAATTAATACGACTCACTATAGGGAGATAGGGGCCATTACGATTATTACTTTCAGATTTAACTCTAAGAGGCATCTTTATTATGTTAATACCTATTAACCAATTACTTAAGAACCCTAACGATATTCCCAAATTACCTTGTGTAACCACCGAGTATCtt                          >  1:492097/1‑300 (MQ=255)`  
                                                                                                                                                                                                                                                                                  `ggAGATTTACCTG‑TGGAGACCGTAACGTATATTGGTCTGGGTCTTTGTGTTCTCGGAGTGTGCCTCATTTCGTGGGGCCTTTGGAACTTAGCCAGAATAATCAAGTCGTTACACGACACTAAGTGATAAACTCAAGGTCCCTAAATTAATACGACTCACTATAGGGAGATAGGGGCCTTTACGATTATTACTTTAAGATTTAACTCTAAGAGGAATCTTTATTATGTTAACACCTATTAACCAATTACTTAATAACCCTAACGATATTCCAGATGTACCTCGTGCAACCGCTGAGTATCTa                          >  1:188508/1‑301 (MQ=255)`  
                                                                                                                                                                                                                                                                                   `agacagtACCTG‑TGGAGACCGTAACGTATATTGGTCTGGGTCTTTGTGTTCTCGGAGTGTGCCTCATTTCGTGGGGCCTTTGGGACTTAGCCAGAATAATCAAGTCGTTACACGACACTAAGTGATAAACTCAAGGTCCCTAAATTAATACGACTCACTATAGGGAGATAGGGGCCTTTACGATTATTACTTTAAGATTTAACTCTAAGAGGAATCTTTATTATGTTAACACCTATTAACCAATTACTTAAGAACCCTAACGATATTCCAGATGTACCTCGTGCAACCGCTGAGTATCTAc                         <  1:130628/295‑1 (MQ=255)`  
                                                                                                                                                                                                                                                                                           `ccTG‑TGGAGACCGTAACGTATATTGGTCTGGGTCTTTGTGTTCTCGGAGTGTGCCTCATTTCGTGGGGCCTTTGTTACTTAGCCAGAATAATCAAGTCGTTACACGACACTAAGTGATAAACTCAAGGTCCCTAAATTAATACGACTCACTATAGGGAGATAGGGGCCTTTACGATTATTACTTTAAGATTTAACTCTAATAGGAATCTTTATTATGTTAACACCTATTAACCAATTACTTAAGAACCCTAACGATATTCCAGATGTACCCCGTGCAACCTCTGTGTATCttcctttctct                 >  1:323227/1‑291 (MQ=255)`  
                                                                                                                                                                                                                                                                                           `ccTG‑TGGAGACCGTAACGTATATTGGTCTGGGTCTTTGTGTTCTCGGAGTGTGCCTCATTTCGTGGGGCCTTTGGGACTTAGCTAGAATAATCAAGTCGTTACACTACACTAAGTGATAAACTCAAGGTCCATAAATTAATACGACTCACTATAGGGAGATAGGGGCAGTTAAGATTATTACTTTAAGATTTAACTCTAAGAGGAATCTTTATTATGTTAACACCTATTAACCAATTACTTAAGAACCATAAAGATATTCAAAATGAACCTCGTGCAACCGCTGAGTATCTACAGGTTAGa                 >  1:456813/1‑301 (MQ=255)`  
                                                                                                                                                                                                                                                                                                  `gagaCCGGAACGTATATTGGTCTGGTTATTTGTGTTCTCTGAGTGTGCCTCATTTCGTGGGGCCTTGGGGAATTAGCCAGAATAATCAAGTCGTTACACGACACTAAGTGATAAACTCAAGGTCCCTAAATTAATACGACTCACTATAGGGAGATAGGGGCCTTTACGATTATTACTTTAAGATTTAACTCTAAGAGGAATCTTTATTATGTTAACACCTATTAACCAATTACTTAAGAACCCTAACGATATTCCAGATGTACCTCGTGCAACCGCTGAGTATCTACAGGTTCGATTAAAc           <  1:109213/301‑1 (MQ=255)`  
                                                                                                                                                                                                                                                                                                  `aagaCTGTAACGTATATTGGTCTGGTTATTTTTGTTCTAGGATTGTGCCTCATTTCGTGGGACCTTTGGGACTTAGCCAGAATAATCAAGTCTATAAACGACACTAAGTGATAAACTCAAGGTCCCTAAATTAATACGACTCACTATAGGGAGATAGGGGCCTTTACGAGTATTACGTTAAGATTTAACTCTAAGATGAATCTTTATTATTTTAACACCTATTAACCAATTACTTAAGAACCCTAACGATATTCCAGATGTACCTCGTGCAACCGCTGAGTATCTACAGGTTCGATTCAAc           <  1:331732/300‑1 (MQ=255)`  
                                                                                                                                                                                                                                                                                                    `gaCCGTAACGTATATTGGTCTGGGTCTTTGTGTTCTCGGAGTGTGCCTCATTTCGTGGGGCCTTTGGGACTTAGCCAGAATAATCAAGTCGTTACACGACACTAATTGATAAACTCAAGGTCCCTAACTTAATACGACTCACTATAGGGAGATAGGGGCCTTTACGATTATTACTTTAAGATTTAACTCTAAGAGGAATCTTTATTATGTTAACACCTATTAACCAATTACTTAAGAACCCTAACGATATTCCAGATGTACCTCGTGCAACCGCTTAGTATCTACAGGTTCGACTCAACtt         >  1:64718/1‑300 (MQ=255)`  
                                                                                                                                                                                                                                                                                                      `ccGTAACGTATATTGGTCTTGGTCTTTGTGTTCTCGGAGTGTGCCTCATTTCGTGGGGCCTTTGGGACTTAGCCCGAATAATCAAGTCGTTACACGACACTAAGTGATAAACTCAAGGTCCCTAAATTAATACGACTCACTATAGGGAGATAGGGGCCTTTACGATTATTACTTTAAGATTTAACTCTATGAGGTATCTTTATTATGTTAACACCTATTAACCAATTACTTAAGAACCCTAACGATCTTCAAGATGTACCCCTTTCAACCGCTTAGTATCTACAGGTTCCATTCAACtttt       >  1:114781/1‑298 (MQ=255)`  
                                                                                                                                                                                                                                                                                                           `gCGTATATTGGTCTGGGACATTGTGTTCTCGGAGTGTGACTAATTTCGTGGGGGCTTTGGGACTTAGCCAGAATAATCAAGTCGTTACACGACACTAAGTGATAAACGCAAGGTCCCTAAAATAAAACGACTCACTATAGGGAGATATGGGCCTTTACGATTAGTACTTTAAGATTTAACTCTAAGAGGAATCTTTATTATGGTAACACCTATTAACCAATTACTTAAGAACCCTAACGAGATTCCAGATGTACCTCGTGCAACCGCTGAGTATCTACAGGTTCGATTCAACTATGCGTAc  <  1:156050/301‑1 (MQ=255)`  

                                                                                                                                                                                                                                                                                                           `|`                                                                                                                                                                                                                                                                                                              
`ATGGTTACTCAGGGATTGCTGGATGGGGTGATACCGCCGAGGACTTCTTGAATAACCCGTTCATAACCGAGCCTAAAACGTCTGTGCTTAAGTCCGGTAAGAACAAAGGCCAAGAGGTTACTAAATGGGTTAAACGCGACCCTGAGCCTCATGAGACGCTTTGGGACTGCATTAAGTCCATTGGCGCGAAGGCTGGTATGACCGAAGAGGATATTATCAAGCAGGGCCAAATGGCTCGAATCCTACGGTTCAACGAGTACAACTTTATTGACAAGGAGATTTACCTG‑TGGAGACCGTAGCGTATATTGGTCTGGGTCTTTGTGTTCTCGGAGTGTGCCTCATTTCGTGGGGCCTTTGGGACTTAGCCAGAATAATCAAGTCGTTACACGACACTAAGTGATAAACTCAAGGTCCCTAAATTAATACGACTCACTATAGGGAGATAGGGGCCTTTACGATTATTACTTTAAGATTTAACTCTAAGAGGAATCTTTATTATGTTAACACCTATTAACCAATTACTTAAGAACCCTAACGATATTCCAGATGTACCTCGTGCAACCGCTGAGTATCTACAGGTTCGATTCAACTATGCGTAC  >  NC_001604.1/18108‑18706`

  

Alignment Legend

Aligned base mismatch/match (shaded by quality score): `ATCG/ATCG` `< 3 ≤` `ATCG/ATCG` `< 10 ≤` `ATCG/ATCG` `< 20 ≤` `ATCG/ATCG` `< 34 ≤` `ATCG/ATCG` `< 38 ≤` `ATCG/ATCG`

Unaligned base: `atcg`    Masked matching base: `atcg`    Alignment gap: `‑`    Deleted base: `‑`
